# Partnering for prevention in under-resourced communities: a randomized pilot study

**DOI:** 10.1186/s12937-022-00824-7

**Published:** 2022-11-25

**Authors:** Angela R. Caldwell, Lauren Terhorst, Jodi S Krall, Danielle W. Thum, Hana R. Uman, Judy L. Dodd, Emily E. Haus, Roxanna M. Bendixen

**Affiliations:** 1 University of Pittsburgh, 4200 Fifth Avenue, Pittsburgh, PA 15260 USA; 2 School of Health and Rehabilitation Sciences Data Center, University of Pittsburgh, 100 Technology Drive, Pittsburgh, PA 15219 USA; 3 UPMC Children’s Hospital of Pittsburgh, 4401 Penn Ave, PA 15224 Pittsburgh, USA; 4 412 Food Rescue, 6140 Station Street, Pittsburgh, PA 15206 USA

## Abstract

**Background:**

Promoting health early in life is crucial to obesity prevention, but families in under-resourced communities face barriers to establishing healthy routines. The purpose of this pilot study was to examine the feasibility and preliminary effects of two dietary interventions for families in under-resourced communities.

**Methods:**

Fifty-one caregivers of young children (aged 0–5 years) were recruited from six community centers located in heavily populated neighborhoods with high poverty rates (i.e., under-resourced neighborhoods) in southwestern Pennsylvania. A longitudinal pilot study was conducted to examine feasibility as a primary outcome and change in dietary variety (24-Hour Recall), risk of nutritional problems (Nutrition Screen for Every Preschooler), and parenting stress (Parenting Stress Index-Short Form) over time and between groups as secondary outcomes. Six sites were randomized to receive Cooking Matters for Parents, Mealtime PREP, or a combined program (Cooking Matters + Mealtime PREP). Cooking Matters for Parents is a six-week nutrition education program designed to help parents of young children plan and cook healthy meals on a budget. Mealtime PREP is a six-week routine-based intervention designed to promote healthy dietary variety among young children. We predicted that we could feasibly deliver both programs and the combined program as determined by a priori benchmarks (ability to recruit ≥ 8 participants per site; achieving an 80% intervention completion rate; being rated as an acceptable intervention by 95% of intervention completers, and treatment fidelity of ≥ 90%). Descriptive statistics and individual growth models were used to analyze data.

**Results:**

Of 51 recruited participants, 49 were eligible, randomized by site, and included in the analyses. Fourteen were randomized to Cooking Matters, 13 to Mealtime PREP, and 22 to the combined program. Three of 4 feasibility benchmarks were met. Over time, improvements were observed in child dietary variety (*p* < 0.01, *SE* = 0.99), child nutrition risk (*p* = 0.01, *SE* = 0.61), and parenting stress (*p* = 0.04, *SE* = 1.33). An additive benefit of the combined intervention was observed for dietary variety *(p* = 0.03, *SE* = 0.79). No adverse events were observed or reported.

**Conclusions:**

Offering complementary dietary interventions in under-resourced communities is feasible and shows promise to improve child and parent health.

**Trial registration:**

ClinicalTrials.gov (NCT03559907).

## Background

Globally, the obesity epidemic has spread dramatically over the last four decades [1, 2]. Children are not spared from this public health crisis, with an estimated 43 million overweight or obese children worldwide [3]. While obesity is preventable, it is influenced heavily by genetics and social factors (e.g., family and physical environment) [4]. The two most significant causal factors in childhood obesity are lack of physical activity and unhealthy eating patterns [5]. Notably, the *Dietary Guidelines for Americans 2020–2025* places an increased emphasis on nutrition for early childhood [6]. Healthy dietary habits, specifically adequate fruit and vegetable consumption, are associated with a lower risk of obesity across the lifespan [7, 8].

Many young children fail to meet the daily recommended intake of fruits and vegetables [9], increasing their risk of obesity [10]. Improving preferences for healthy food early in life is crucial, as childhood preferences influence eating behaviors in adulthood [11]. Families in low-income neighborhoods face barriers to developing healthy habits such as time and resource constraints and food-related values and beliefs (e.g., cultural values, body image, pressure to eat socially) [12]. Approximately 15% of preschoolers from low-income families are classified as obese before their fifth birthday, as compared with the 8% observed in the general population [13]. Children in under-resourced communities, defined as population-dense metropolitan areas with high poverty rates [14], are more likely to become obese at a younger age than peers [13]. Interventions that facilitate the development of healthy habits by coaching caregivers to build healthier routines early in life are needed to combat childhood obesity [15].

In response, Cooking Matters (CM), a program of 6 weekly sessions focused on meal planning, cooking, and purchasing healthy food on a budget, was developed to reduce obesity risk among low-income individuals [16]. Specific to families, CM for Parents is specialized in providing nutrition recommendations and food preparation strategies for young children [17]. CM, when offered to small groups of 8–15 participants, has improved confidence in food management and increased home-cooked meals, but changes in child dietary variety are unclear [18]. Supplementing CM with an intervention to improve child acceptance of healthy foods has not been evaluated.

Promoting Routines of Exploration and Play during Mealtime (Mealtime PREP) was developed by a pediatric occupational therapist to improve young children’s dietary variety and mealtime behavior. This program aims to empower caregivers to build predictable routines for family meals and provides opportunities for food exploration and play. Mealtime PREP has been shown to improve mealtime behavior and nutritional risk for young children with sensory food aversions in the home [19, 20]. The effects of delivering this intervention to groups of participants in under-resourced neighborhoods are unknown. Offering complementary programs (e.g., Mealtime PREP and CM for Parents) may provide a cost-effective approach to promoting health.

The purpose of the current study was to 1) assess the feasibility of offering Cooking Matters for Parents and/or Mealtime PREP to families in under-resourced neighborhoods, and 2) examine the independent and additive effects of the programs on child dietary variety and risk of nutritional problems (e.g., limited dietary variety, obesity), and caregiver stress.

## Methods

Caregiver participants were recruited between September 2018 and June 2019 from 6 Family Support Centers (FSCs) in under-resourced, urban, and suburban neighborhoods in Western Pennsylvania. The FSCs provide free, holistic home visiting and center-based interdisciplinary services for infants, children, and families living in communities that are heavily populated and experience high rates of poverty [21]. Using a partner-led strategy, FSC leaders headed recruitment efforts. Inclusion criteria were (1) caregiver of a child aged 0–5 years, (2) fluent in English, (3) willingness to participate in sessions. Participants were excluded if they had completed either program in the past. All participants provided written informed consent. This study was reviewed and approved by the University’s Institutional Review Board and is registered on ClinicalTrials.gov (NCT03559907).

This randomized longitudinal pilot study consisted of 3 treatment conditions: (1) CM; (2) Mealtime PREP; or (3) Combined: CM + Mealtime PREP. Prior to recruitment, six sites were identified to host a group based on prior success engaging members in similar programs. The randomization scheme was designed by a biostatistician using SAS PROC PLAN, and sites were randomized in blocks of 3 to ensure two sites (i.e., groups) were assigned to each treatment condition. Based on prior recruitment for community programming and attrition rates, we aimed to recruit 8 participants per site. An a priori power analysis determined that with a sample size of 48, we would be 80% powered to detect within-between interaction effects and between-group effects with an alpha of 0.05. Leaders recruited fifty-one caregivers within each FSC, but two were ineligible due to child age. Therefore, 49 eligible participants were included in our analyses. Assessments were caregiver-reported and occurred at baseline, directly following intervention (6 to 12 weeks after baseline), and approximately three months after intervention completion; compensation for each assessment session increased over time ($25, $50, $75). Incentives to boost attendance at group sessions included weekly groceries and a small appliance (e.g., blender, toaster oven) for attending ≥ 4 of 6 sessions for each program offered. All study-related activities (assessments and group sessions) occurred at the local FSCs and were scheduled on days and times identified by FSC leaders as convenient for members of their center. Trained graduate student research assistants collected assessment data using paper forms or the Research Electronic Data Capture (REDCap) mobile application on an iPad. Research assistants were available to help participants interpret questions as needed and were not blinded to intervention as measures were solely based on caregiver report and, therefore, unlikely to be influenced by bias related to the assessor’s knowledge of group assignment.

### Interventions

CM for Parents was led by trained instructors and included six weekly, 2-h sessions to increase self-sufficiency in the kitchen. Each session was held in the participants’ local FSC and included meal preparation, didactic teaching, and sharing a meal as a group. Participants learned skills related to nutrition, healthy meal preparation, and cost-effective purchasing of healthy foods. Participants received groceries each week and practiced cooking skills within their homes. This course did not address child mealtime behavior, but healthy, child-friendly foods were discussed. Instructors had experience in nutrition education and culinary skills and completed seven hours of training to follow the manual of procedures using a gold standard checklist for each session [16].

Mealtime PREP was also delivered in the participants’ local FSC and led by a pediatric occupational therapist. It included 6-weekly, 2-h sessions to enhance daily child meals to promote healthy dietary variety. This intervention features a behavioral activation approach to facilitate the adoption of new, healthy family mealtime routines [19]. Caregivers selected healthy foods (e.g., vegetables, lean proteins, fruits) frequently offered in their homes to practice skills between sessions. Strategies to improve acceptance of healthy foods included: (1) frequent family meals; (2) positive reinforcement (i.e., verbal praise and positive attention); (3) repeated exposure; and (4) play to increase interaction [22–24]. Each week, participants received groceries and child-friendly dishware to promote proper portions (e.g., divided plate and measuring cups) or serving ware for family-style meals. The instructor completed 8 h of training and followed a manual of procedures. During each session, approximately 15 min were set aside to troubleshoot solutions to unique family circumstances (e.g., new baby in the home, scheduling conflicts, feeding problems) with individual participants.

Participants recruited to either of the two sites randomized to the Combined treatment received both programs in sequence, CM followed by Mealtime PREP, at their local FSC. This group received 12-weekly, 2-h treatment sessions with the same trained staff and established protocols.

### Feasibility outcomes

Feasibility benchmarks were our primary outcome measures based on programmatic recommendations and preliminary data. They included: (1) Ability to recruit, on average, > 8 participants per site; (2) achieving an 80% intervention completion rate; (3) being rated as an acceptable intervention by 95% of intervention completers, and (4) treatment fidelity of ≥ 90% for CM and Mealtime PREP regardless of treatment condition. The intervention completion rate is the percentage of participants attending ≥ 4 of 6 sessions for each program. Therefore, a participant in the Combined treatment would need to complete at least four sessions of each program to meet this benchmark. Intervention acceptance was assessed using the Treatment Acceptability Questionnaire (TAQ), a validated eight-item Likert-type scale [25]. Participants completed the TAQ directly after the intervention, and a score ≥ 28 was used as a cut-off for intervention acceptability [19]. Treatment fidelity was assessed via video review of sessions and completion of checklists created using each program’s manual and established gold standards for CM [16].

### Preliminary effects: intervention outcome

Data were collected on secondary outcomes of child dietary variety, child risk of nutritional problems, caregiver stress, and participant characteristics at baseline, post-intervention, and a 3-month follow-up. All data were collected in person at the FSCs apart from the 3-Day Food Diary, collected via mail.

Data on dietary variety were collected using two methods. Participants were provided with a 3-Day Food Diary and instructed to record all foods their child consumed over three days, including approximate amounts. They were asked to include at least one weekend day and weekday and given a self-addressed stamped envelope to return the diary. A 24-h Dietary Recall was collected at each time point, and the number of unique foods consumed each day was tallied.

The Nutrition Screening for Every Preschooler (NutriSTEP) assessed the risk of nutrition-related problems. This 17-item, caregiver-reported, multiple-choice assessment is a valid and reliable screen for risk of nutritional problems (e.g., obesity, limited dietary variety) in young children [26]. Questions cover food frequency, mealtime practices, screen time, and child growth.

The Parenting Stress Index, Short-Form (PSI-SF) assessed overall caregiver stress level. This reliable tool has been validated in diverse populations, and percentile scores ≥ 90% represent clinically significant caregiver stress [27]. Higher scores represent higher stress, with the total score representing the overall stress level experienced within the caregiver role [28].

Participant characteristics (e.g., gender, age, ethnicity) and caregiver education, employment, household income, and total number of siblings were collected using a demographic form. Race was self-reported by the caregivers of the children from a list including White, Black/African American, Asian, American Indian/Alaskan Native, Native Hawaiian/Pacific Islander, or Other (specify).

### Data analysis

Descriptive statistics (e.g., means, frequencies, percentages) were used to determine whether feasibility benchmarks were met. Prior to modeling, detailed exploratory analyses were performed, including missing data analysis and screening for anomalies. Potential imbalances between the two treatment groups (combined vs. independent) were examined using chi-square analyses for categorical variables and t-tests or Mann–Whitney U tests for continuous variables as appropriate. Associations between the dependent variables at baseline and potential demographic confounders (e.g., race, education, employment status) were calculated.

A series of individual growth models were fitted to examine treatment effects over time. Mixed-effects models were used as they allow both fixed and random effects and modeling with missing outcome data, so long as they are missing at random [29]. For each dependent variable (dietary variety, risk of nutritional problems, total caregiver stress), an unconditional model was run with a random participant effect to examine change in the entire sample over time. Parameter estimates and measures of model fit (i.e., Akaike Information Criterion and Bayesian Criterion) were examined. Next, a conditional model was fitted, examining interaction and main effects over time. Potential covariates were identified based on significant correlations with outcome measures at baseline (i.e., employment status and education level) or likelihood to impact outcomes (i.e., intervention completion) and added to the best fitting model. An independent covariance structure with group and time considered as fixed effects, and a random effect for participants was used in all models. Differences in treatment group (combined vs independent) as well as condition (CM vs. Mealtime PREP vs Combined) were examined. Post hoc tests were performed for significant interaction or main effects. Statistical analyses were conducted with Stata SE (v.16) using an alpha of 0.05.

## Results

**Fig. 1:**
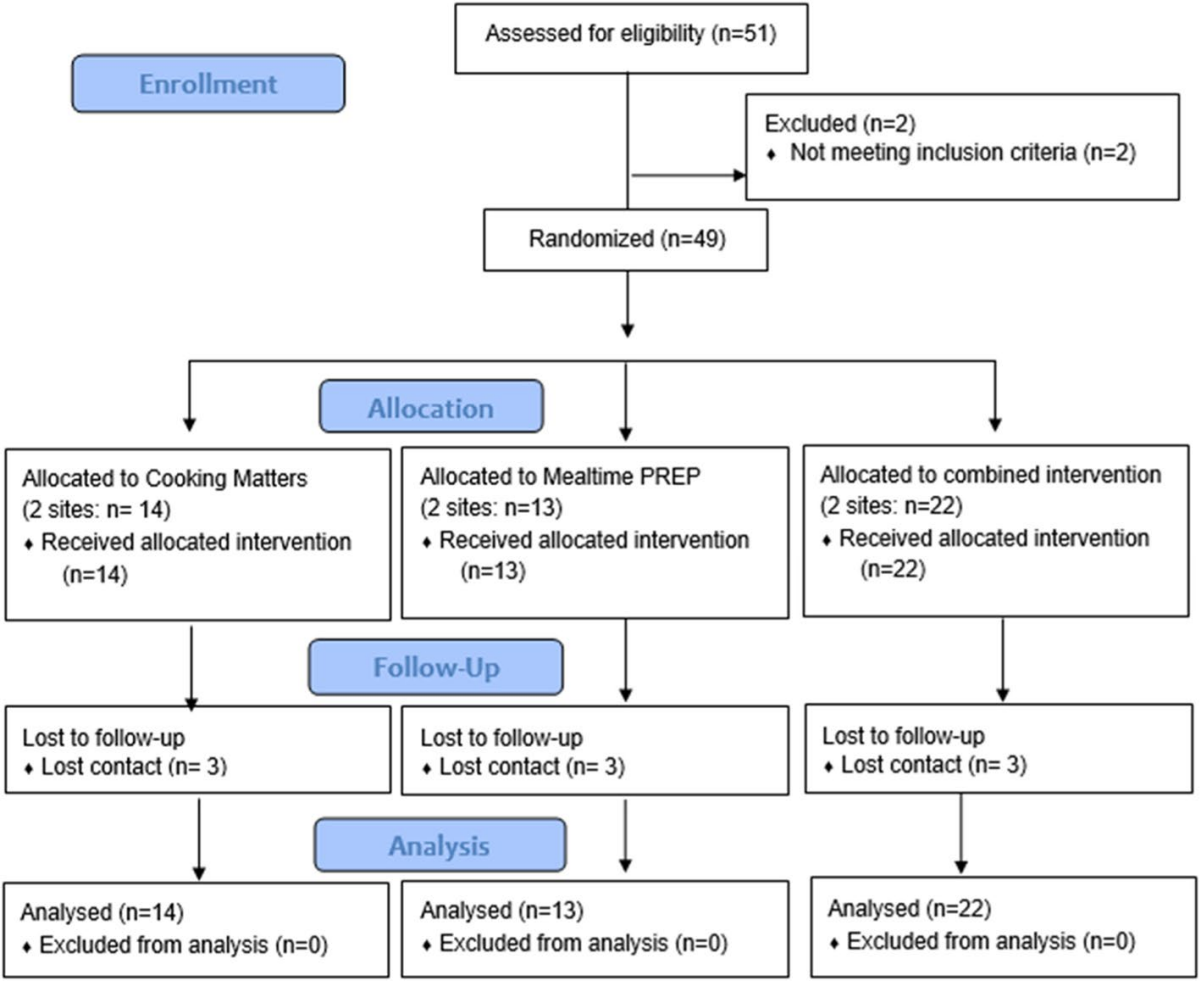
CONSORT flow diagram

The number of participants recruited per site ranged from 6 to 13. Between 2 sites per treatment condition, 14 were randomized to CM, 13 to Mealtime PREP, and 22 to the Combined program, and all were included in the mixed effects modeling (Fig. 1). Of the 49 caregiver participants, 94% identified as female, 55% as Black/African American, and 71% as Non-Hispanic. Caregiver participants were primarily mothers (88%) and included three grandmothers (6%) and three fathers (6%). Most participants were single (78%) and reported their highest level of education as high school (69%) and a household income of less than $20,000 per year (61%). Just over half of the children were female (51%), and most were Black/African American (53%) and Non-Hispanic (69%). Of note, approximately one-fourth (24%) of these children had received early intervention services; specific diagnoses reported were Attention Deficit Hyperactivity Disorder, Down syndrome, and Shaken Baby Syndrome. No significant differences in demographic features or baseline scores were identified between groups (Table 1). No adverse effects were observed or reported.

**Table 1:** Sociodemographic Characteristics (*N* = 49)

	Independent: Cooking Matters OR Mealtime PREP (*N* = 27)		Combined: Cooking Matters + Mealtime PREP (*N* = 22)	
**Characteristic**	**N**	**%**	**N**	**%**
Caregiver Gender				
Female	25	92.6	21	95.5
Male	2	7.4	1	4.5
Child Gender				
Female	12	44.4	13	59.0
Male	15	55.5	9	40.9
Caregiver Race				
Black/African American	16	59.3	11	50.0
White	4	14.8	7	31.8
Multiracial	3	11.1	2	9.1
Other/Not reported	4	14.8	2	9.1
Child Race				
Black/African American	14	51.9	12	54.5
Multiracial	8	29.6	2	9.1
White	2	7.4	6	27.3
Other/Not reported	3	11.1	2	9.1
Caregiver Ethnicity				
Hispanic	4	14.8	2	9.1
Non-Hispanic	18	66.7	17	77.3
Not reported	5	18.5	3	13.6
Child Ethnicity				
Hispanic	4	14.8	2	9.1
Non-Hispanic	17	63.0	17	77.3
Not Reported	6	22.2	3	13.6
Caregiver Highest Education				
No Highschool Diploma	3	11.1	0	0
Highschool Diploma	18	81.8	16	72.7
Vocational Degree	3	11.1	3	13.6
Associate degree	0	0	1	4.5
Bachelor’s Degree	3	11.1	2	9.1
Caregiver Employment Status				
Full-time	3	11.1	3	13.6
Part-time	3	11.1	5	22.7
Works in home	2	7.4	3	13.6
Not employed	11	40.7	8	36.4
Unable to work	7	25.9	3	13.6
Retired	1	3.7	0	0
Household Income ($)				
0–20,000	18	66.7	12	54.5
20,000–40,000	3	11.1	5	22.7
40,000–60,000	1	3.7	0	0
60,000–80,000	1	3.7	2	9.1
80,000–100,000	1	3.7	0	0
Not reported	4	14.8	3	13.6
Number of Siblings				
0 (only child)	8	30	2	9
1 – 2	9	33	16	73
3 +	8	30	4	18
Caregiver age in years: M (SD)	35.5 (11.1)		34.3 (10.5)	
Child age in months: M (SD)	40.2 (18.7)		40.0 (16.2)	

*M* Mean, *SD* Standard deviation, *N* Number

Three of 4 feasibility benchmarks were met or met partially. Thirty-three of 49 participants (67%) achieved intervention completion, with 80% (39) participating in data collection throughout the study. On average, 8 participants were recruited per group, but three groups did not meet this mark. Both interventions achieved acceptability as 95% of participants scored ≥ 28 on the TAQ (*M* = 39.51, *SD* = 1.20). Treatment fidelity was excellent; CM and Mealtime PREP instructors met nearly all protocol standards (98 and 99%, respectively).

### Preliminary effects: intervention outcomes

**Fig. 2:**
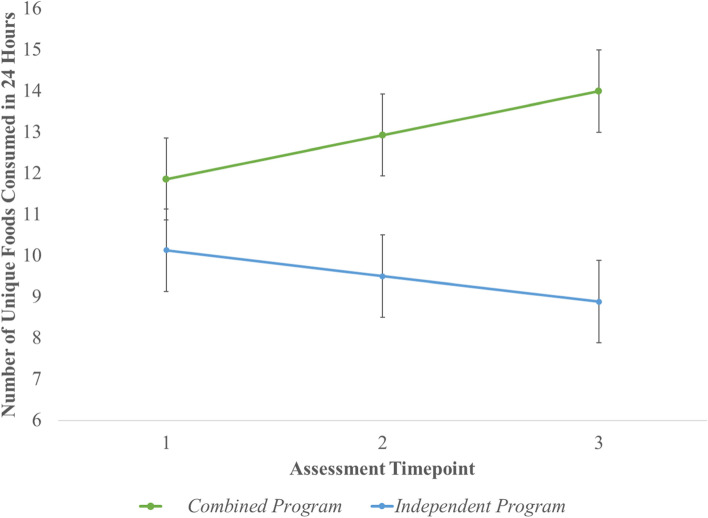
Child dietary variety over time

Dietary variety could not be examined using the 3-Day Food Diary due to low return rate at each assessment timepoint (10, 2, and 3, respectively). Using data from 24-h Food Recalls, likelihood ratio tests determined that a model with the main and interaction effects demonstrated better fit than an unconditional model with time only (*p* < 0.05). Models examining differences between the three treatment conditions (Mealtime PREP, CM, Combined) did not yield significant differences between independent programs and did not demonstrate a better model fit than those examining differences between groups (combined vs. independent). Caregivers reported that at baseline, their children consumed, on average, 11 unique foods in a day. On average, children whose caregivers participated in the combined program demonstrated a gain of 1.7 more unique foods per time point than those whose caregivers participated in either program in isolation (*p* = 0.03, *SE* = 0.79; see Fig. 2). Post-hoc tests revealed greater dietary variety for the combined group after intervention (*p* = 0.01, *M*_*difference*_ = 3.42, *SE* = 1.25), with the combined group performing significantly better at follow-up as well (*p* = 0.001, *M*_*difference*_ = 5.12, *SE* = 1.55; Table 2).

**Table 2:** Outcomes by treatment group and time after controlling for caregiver education, employment, and intervention completion

Outcome	Baseline	Post-Intervention	3-month Follow-up
Child Dietary Variety			
Combined Program	11.86 (1.04)	12.93 (0.91)*	13.99 (1.12)*
Cooking Matters OR Mealtime PREP	10.13 (0.97)	9.50 (0.84)*	8.88 (1.04)*
Child Risk of Nutritional Problems^+^			
Combined Program	19.37 (1.20)*	19.73 (1.08)	20.09 (1.29)
Cooking Matters OR Mealtime PREP	22.48 (1.09)*	20.92 (0.98)	19.36 (1.20)
Caregiver Stress^+^			
Combined Program	77.18 (5.01)	73.74 (4.73)	70.30 (5.25)
Cooking Matters OR Mealtime PREP	77.14 (4.54)	74.46 (4.25)	71.78 (4.81)

Reported as least square means with standard errors in parentheses

*PREP* Promoting Routines of Exploration and Play

Significant difference (*p* < .05) between groups indicated by an asterisk (*) and over time for the entire sample indicated by a cross ( +). Higher scores represent better dietary variety, higher risk of nutritional problems, and higher caregiver stress

Similarly, a series of models were fitted to examine changes in NutriSTEP scores over time. The models including the interaction of time and receiving a combined versus an independent program demonstrated better model fit than the unconditional model (*p* = 0.08). According to the full model with covariates, the average score on the NutriSTEP at baseline was 23, representing a moderate risk of nutritional problems. Scores for the entire sample decreased by 1.5 points at each assessment timepoint, on average (*p* = 0.01, *SE* = 0.61), indicating reduced risk over time (Table 2). There was a significant interaction between group and time (*p* = 0.02) and a significant main effect for group (*p* = 0.01). Post hoc analyses revealed that significant differences between groups were only present at baseline (*p* = 0.05), with the combined group scoring significantly lower than the group that received one program (*p* = 0.05, *M*_*difference*_ = 3.11, *SE* = 1.60).

Models were fitted as above to examine interactions between treatment conditions of group and time and the main effects over time for caregiver stress. No significant interactions were found in any models, and the models including interactions and covariates did not improve model fit. In the unconditional model, a main effect for time (*p* = 0.04) indicated that participant stress decreased by 2.7 points (*SE* = 1.33) on average at each time point (Table 2).

## Discussion

Study findings indicate that it is feasible to pair complementary programs to promote health early in life for families of young children in under-resourced neighborhoods. Participants rated both interventions as acceptable, and instructors demonstrated excellent fidelity to intervention protocols. The overall recruitment goal was met for total participants per group; however, this goal was only achieved by 50% of sites. Recruitment barriers varied by site and included issues such as recent change in the physical location of the FSC and scheduling conflicts. Sessions were scheduled at convenient times for members of each FSC, as identified by center leaders; nevertheless, some participants faced barriers related to work, school, and service commitments. Supplementing recruitment efforts using outreach to nearby childcare centers is another effective strategy that could be used in future studies [30]. Overall, participant retention and intervention completion rates represented success and were higher than in similar studies [18, 31]. Offering health-promoting interventions in under-resourced neighborhoods may minimize barriers associated with transportation and time constraints. In addition to the strategies used in this study (incentives, personal approach to programming, participant convenience), frequent check-ins with participants could boost sustained engagement in future programs [30].

Observed improvements in child dietary variety, child risk of nutritional problems, and caregiver stress are promising. On average, participants receiving the combined program demonstrated minimal risk for nutritional problems at baseline and remained stable throughout study participation [26]. However, on average, the group that received an independent intervention demonstrated moderate risk of nutritional problems at baseline and a clinically relevant shift to minimal risk by the study end [26]. This suggests that both interventions can potentially decrease the risk of nutrition-related problems for children with higher risk at baseline.

Participants who received the combined program demonstrated improvements in dietary variety. Importantly, dietary quality was not assessed; the number of unique foods consumed was tallied as an indicator of a child’s willingness to try a variety of foods. This finding indicates that complementary skills gained by participating in both programs may yield an additive benefit for child dietary variety. Finally, this study supports a growing body of evidence that caregiver stress is related to social support [32, 33].

Limitations of this study include a relatively small sample size with unequal groups, which represents a threat to external validity and the potential for a Type II error. Participants in the combined group received more sessions; therefore, dosage may have influenced outcomes and warrants additional investigation. Additionally, reliance on caregiver-reported measures threatens internal validity, increasing the risk of response bias. It is unclear why the return rate for 3-Day Food Diaries was low even though self-addressed stamped envelopes were provided to participants. We predict that caregiver education, competing demands, and the high participant burden associated with keeping daily food logs may have presented challenges to data collection using the Food Diary. For future trials, we are exploring more user-friendly methods of assessing dietary variety (using pictures or mobile apps). Additionally, innovative strategies, such as frequent discussions about barriers to participation with ongoing problem-solving, may be needed to promote intervention completion among residents of under-resourced neighborhoods [34].

This pilot study provides data to justify the need for larger, confirmatory studies to determine the long-term effects of offering CM and Mealtime PREP in combination. Study strengths include high treatment fidelity and reproducibility using manualized intervention programs. Furthermore, evidence-based strategies to improve engagement achieved high participant retention rates and intervention acceptability. A diverse sample of participants strengthens the external validity. More data are needed to make causal inferences and determine the effectiveness of these dietary programs.

## Conclusions

In conclusion, offering manualized health-promoting interventions in under-resourced neighborhoods is feasible and was widely accepted across sites. Delivering these programs in convenient locations, offering incentives, and collaborating with established community leaders are promising strategies to engage low-income and minority participants. Preliminary results indicate that these programs may yield improvements in child dietary variety, child risk of nutritional problems, and caregiver stress. Additional research is needed to determine the best combination of programming to promote health among children at risk.

## Data Availability

The datasets used and/or analyzed during this study are available from the corresponding author on reasonable request and pending a data use agreement.

## Abbreviations

CM: Cooking Matters
FSC: Family Support Center
Mealtime PREP: Promoting Routines of Exploration and Play during Mealtime
NutriSTEP: Nutrition Screening Tool for Every Preschooler
PSI-SF: Parenting Stress Index-Short Form
REDCap: Research Electronic Data Capture
TAQ: Treatment Acceptability Questionnaire

## References

[1] De Onis M, Blössner M, Borghi E (2010). Global prevalence and trends of overweight and obesity among preschool children. Am J Clin Nutr.

[2] Güngör NK (2014). Overweight and obesity in children and adolescents. J Clin Res Pediatr Endocrinol.

[3] Ogden CL, Carroll MD, Kit BK, Flegal KM (2014). Prevalence of childhood and adult obesity in the United States, 2011–2012. JAMA.

[4] Ross SE, Flynn JI, Pate RR (2016). What is really causing the obesity epidemic? A review of reviews in children and adults. J Sports Sci.

[5] Han JC, Lawlor DA, Kimm SYS (2010). Childhood obesity. Lancet.

[6] U.S. Department of Health and Human Services and U.S. Department of Agriculture. 2015–2020 Dietary Guidelines for Americans, 8th edition. 2015. Available from: https://www.dietaryguidelines.gov/sites/default/files/2020-12/Dietary_Guidelines_for_Americans_2020-2025.pdf.

[7] Rautiainen S, Wang L, Lee I-M, Manson JE, Buring JE, Sesso HD (2015). Higher intake of fruit, but not vegetables or fiber, at baseline is associated with lower risk of becoming overweight or obese in middle-aged and older women of normal BMI at baseline. J Nutr.

[8] Wang X, Ouyang Y, Liu J, Zhu M, Zhao G, Bao W (2014). Fruit and vegetable consumption and mortality from all causes, cardiovascular disease, and cancer: systematic review and dose-response meta-analysis of prospective cohort studies. BMJ.

[9] Siega-Riz AM, Deming DM, Reidy KC, Fox MK, Condon E, Briefel RR (2010). Food consumption patterns of infants and toddlers: where are we now?. J Amer Diet Assoc.

[10] Angelino D, Godos J, Ghelfi F, Tieri M, Titta L, Lafranconi A (2019). Fruit and vegetable consumption and health outcomes: an umbrella review of observational studies. Int J Food Sci Nutr.

[11] Mascola AJ, Bryson SW, Agras WS (2010). Picky eating during childhood: a longitudinal study to age 11 years. Eat Behav.

[12] Baruth M, Sharpe PA, Parra-Medina D, Wilcox S (2014). Perceived barriers to exercise and healthy eating among women from disadvantaged neighborhoods: results from a focus groups assessment. Women Health.

[13] Cunningham SA, Kramer MR, Narayan KMV (2014). Incidence of childhood obesity in the United States. New Engl J Med.

[14] Eberhardt P, Wial H, Yee D. The new face of under-resourced communities. Available at SSRN 3796471. 2020. Available from: https://icic.org/wp-content/uploads/2020/10/The-New-Face-of_Under-Resourced-Communities.pdf.

[15] Smith JD, St George SM, Prado G (2017). Family-Centered Positive Behavior Support Interventions in Early Childhood To Prevent Obesity. Child Dev.

[16] Cooking Matters. Cooking Matters gold standards for instructors. 2018. Available from: https://s14621.pcdn.co/wp-content/uploads/2019/04/Volunteer-Gold-Standards.pdf.

[17] Cooking Matters. Who we are. 2019. Available from: http://cookingmatters.org/who-we-are.

[18] Pooler JA, Morgan RE, Wong K, Wilkin MK, Blitstein JL (2017). Cooking matters for adults improves food resource management skills and self-confidence among low-income participants. J Nutr Educ Behav.

[19] Caldwell AR, Skidmore ER, Raina K, Rogers JC, Terhorst L, Danford CA, et al. A behavioral activation approach to parent training: feasibility of “Promoting Routines of Exploration and Play during Mealtime.” Am J Occup Ther. 2018;72:1–8. 10.5014/ajot.2018.028365 30760395

[20] Caldwell AR, Skidmore ER, Bendixen RM, Terhorst L. Examining child mealtime behavior as parents are coached to implement the Mealtime PREP intervention in the home: findings from a pilot study. Br J Occup Ther. 2020;83(10):631–7. 10.1177/0308022620920086 PMC10254738 37304357

[21] UPMC. Family and social needs support. 2020. Available from: https://www.chp.edu/our-services/community-health/social-needs-support.

[22] Remington A, Anez E, Croker H, Wardle J, Cooke L (2012). Increasing food acceptance in the home setting: a randomized controlled trial of parent-administered taste exposure with incentives. Am J Clin Nutr.

[23] Cooke LJ, Chambers LC, Añez EV, Wardle J (2011). Facilitating or undermining? The effect of reward on food acceptance. A narrative review. Appetite.

[24] Sweetman C, McGowan L, Croker H, Cooke L. Characteristics of family mealtimes affecting children’s vegetable consumption and liking. J Amer Diet Assoc. 2011;111(2):269–73. 10.1016/j.jada.2010.10.050 21272701

[25] Krain AL, Kendall PC, Power TJ (2005). The role of treatment acceptability in the initiation of treatment for ADHD. J Atten Disord.

[26] Simpson JAR, Keller HH, Rysdale LA, Beyers JE (2008). Nutrition screening tool for every preschooler (NutriSTEP™): Validation and test–retest reliability of a parent-administered questionnaire assessing nutrition risk of preschoolers. Eur J Clin Nutr.

[27] Whiteside-Mansell L, Ayoub C, McKelvey L, Faldowski RA, Hart A, Shears J (2007). Parenting stress of low-income parents of toddlers and preschoolers: psychometric properties of a short form of the parenting stress index. Parent Sci Pract.

[28] Abidin RR (2012). Parenting stress index.

[29] Ibrahim JG, Molenberghs G (2009). Missing data methods in longitudinal studies: a review. TEST.

[30] Nicholson LM, Schwirian PM, Klein EG, Skybo T, Murray-Johnson L, Eneli I (2011). Recruitment and retention strategies in longitudinal clinical studies with low-income populations. Contemp Clin Trials.

[31] Otterbach L, Mena NZ, Greene G, Redding CA, De Groot A, Tovar A (2018). Community-based childhood obesity prevention intervention for parents improves health behaviors and food parenting practices among Hispanic, low-income parents. BMC Obes.

[32] Kang NR, Kim DH, Kwack YS (2019). The effect of community-based parent education program on parenting stress according to adult attachment styles. J Kor Acad Child Adolesc Psychiatr.

[33] Gleeson JP, Hsieh C-M, Cryer-Coupet Q (2016). Social support, family competence, and informal kinship caregiver parenting stress: the mediating and moderating effects of family resources. Child Youth Serv Rev.

[34] Zweben A, Fucito LM, O’Malley SS. Effective strategies for maintaining research participation in clinical trials. Drug Inf J. 2009;43(4):459–67. 10.1177/009286150904300411 PMC3848036 24311825

